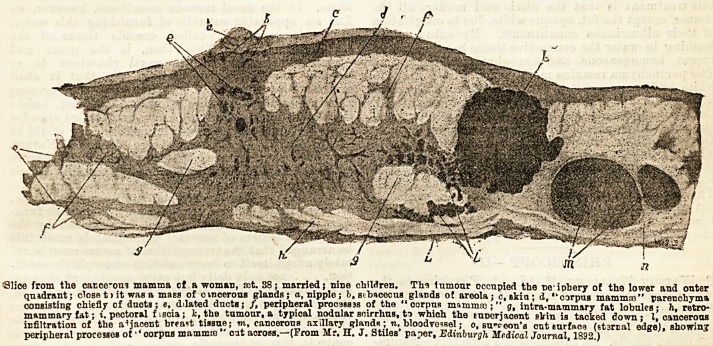# Some Points in the Treatment of Scirrhus of the Breast

**Published:** 1893-05-20

**Authors:** 


### THE HOSPITAL CLINIC.

[*The Editor will be glad to receive offers of co-operation and contributions from members of the profession All letters should be addressed to* THE EDITOR, THE LODGE, PORCHESTER SQUARE, LONDON, W.]

### ROYAL INFIRMARY, EDINBURGH.

### SOME POINTS IN THE TREATMENT OF SCIRRHUS OF THE BREAST.

Any surgical operation to be rational and scientific must be founded on an accurate knowledge of anatomy, and of the pathology of the condition for which it is undertaken. This is especially true of malignant affections, in the widest sense of the term malignant, including all conditions in which any part left behind means certainty of return of the disease. Perhaps the commonest examples of this are excisions of joints for tubercular disease and the removal of the breast for scirrhus carcinoma. An important investigation recently carried out by Mr. Harold J. Stiles, in the surgical laboratory of the University of Edinburgh, and in the wards of the Royal Infirmary, had for its object the elucidation of some of the undecided points in relation to the latter disease. Some of the practical results obtained are embodied in this contribution.

*Surgical Anatomy.*—Mr. Stiles points out that the anatomy of the breast, as described in the ordinary text books, does not meet the requirements of the surgeon, inasmuch as the limits of the gland are made to appear definitely fixed and circumscribed, as if the breast were a completely encapsulated organ. There is no capsule, properly so-called, to the mamma.

The parenchyma is irregularly broken up towards the periphery, and becomes intimately blended with the surrounding connective tissue and subcutaneous fascia. For clinical purposes, Mr. Stiles describes the breast as extending vertically "from the lower border of the second rib, to the sixth costal cartilage at the angle, where it begins to sweep upwards to the sternum;" and horizontally "from a little within the edge of the sternum opposite the fourth rib or interspace, to the fifth rib or interspace opposite the mid-axillary line." He emphasises the importance of remembering Spence's "axillary tail," which passes into the axilla as high as the third rib, between the pectoralis major and serratus magnus muscles.

In studying the anatomy of the breast from the point of view of the operating surgeon, there must be taken into account—(1) The general mass of the gland, the corpus mammæ; (2) the outlying peripheral processes; (3) the retro-mammary tissue and pectoral fascia; and (4) the lymphatic system of the breast.

With regard to the corpus mammæ (*d*) there is little room for surgical error, as it is a compact, visible and tangible mass, varying only slightly in size in different individuals; but it is far otherwise with the peripheral processes (*f*) which radiate in every direction from the corpus. These pass forwards in the meshes of the fine fibrous bands, the ligaments of Cooper, and gain an attachment to the corium of the skin (*c*). They also extend freely into the surrounding sub-cutaneous fat and areolar tissue. Posteriorly they assume still greater importance by freely infiltrating the retro-mammary tissue (*h*) piercing even the pectoral fascia (*i*).

The clinical importance of the lymphatic system of the breast lies in the fact that it is by means of it that the cancerous disease spreads, not only throughout the organ itself, but also to the neighbouring lymphatic glands, and thence to other organs. Microscopically, Mr. Stiles has been able to distinguish several distinct series of lymphatic channels at different levels in the breast, all freely inter-communicating and ultimately opening into large trunks which pierce the deep fascia and discharge themselves, for the most part into the axillary, and to a less extent into the sternal glands. He says, "there is no doubt that the lymphatics of the two breasts communicate to a certain extent through a median anastomosis," a point which bears directly on the consecutive affection of both breasts.

Slice from the cancerous mamma of a woman, æt. 38; married; nine children. The tumour occupied the periphery of the lower and outer quadrant; close to it was a mass of cancerous glands; *a*, nipple; *b*, sebaceous glands of areola; *c*, skin; *d*, "corpus mammæ" parenchyma consisting chiefly of ducts; *e*, dilated ducts; *f*, peripheral processes of the "corpus mammæ;" *g*, intra-mammary fat lobules; *h*, retro-mammary fat; *i*, pectoral fascia; *k*, the tumour, a typical nodular scirrhus, to which the superjacent skin is tacked down; *l*, cancerous infiltration of the adjacent breast tissue; *m*, cancerous axillary glands; *n*, bloodvessel; *o*, surgeon's cut surface (sternal edge), showing peripheral processes of "corpus mammæ" cut across.—(From Mr. H. J. Stiles' paper, *Edinburgh Medical Journal*, 1892.)

*Pathology.*—The axillary glands are very numerous, as many as thirty being sometimes removed in clearing the axilla at operation. Many of these show the characteristic cancerous condition, others are fattily degenerated, while some remain normal.

It is important to note that not only may cancerous foci be found in the breast itself, and in the axillary glands, but in the intermediate lymphatic vessels masses of cancer cells are often to be found.

The microscopic pathology, as described by Mr. Stiles, is of great interest and importance, but suffice it to say here regarding it that he has been able to demonstrate minute cancerous emboli in situations far removed from the main primary mass, *e.g.*, in the circum-mammary fat, the ligaments of Cooper, the pectoral fascia, and elsewhere, all indicating the radical nature of an operation which aims at complete removal.

*Practical Deductions.*—Accepting these facts in the light of our clinical experience of the operations for mammary cancer, it becomes obvious that the explanation of the too frequent recurrence of the disease is to be found in the comparatively limited procedures usually carried out.

To afford any hope of immunity from recurrence the whole breast, including Spence's axillary tail, the whole of the lymphatic glands and fat of the axilla, and the intermediate lymph vessels must be removed. Not only so, but the whole of the pectoral fascia, often a portion of the subjacent muscle, and always a wide area of skin must be sacrificed.

*Nitric Acid Test.*—To aid in rapidly distinguishing between cancerous and non-cancerous tissue during an operation, Mr. Stiles employs a test—for convenience called the "Nitric Acid Test"—which is as follows: Immediately the breast is removed all the blood is washed off it, and the mass is placed in one or two pints of a 5 per cent. solution of nitric acid for about ten minutes, after which it is washed in running water for three or four minutes. "The rationale of this treatment is that the nitric acid renders all the tissues, except the fat, opaque white, due to coagulation of their albuminous constituents. By subsequently washing in water the connective tissue becomes translucent, homogeneous, and somewhat gelatinous. . . . The parenchyma remains more or less dull, greyish-white, and opaque, due to the coagulation of the more highly albuminous epithelial cells. The fat is unaltered. Cancerous tissue behaves in the same way as the parenchyma, and is rendered even denser and more opaque. In very cellular cancers the tissue resembles boiled white of egg, though of a greyish colour. The characteristic arrangement of the parenchyma is generally sufficient to distinguish it from the cancerous tissue." This test often reveals nodules of cancer on the surgeon's cut surface, showing that the whole disease has not been removed, and indicating the necessity for immediately enlarging the incision in the appropriate direction.

## Figures and Tables

**Figure f1:**